# A Managing Wife

**Published:** 1855-01

**Authors:** 


### A MANAGING WIFE.

Some women are never happy unless when they are scrubbing, brushing, sweeping or otherwise toiling in household affairs, although they have servants to do all that they require. The Hon. Henry Erskine's first wife was one of this class, and her extreme nervous irritability and eccentric ways, it may be supposed, did not contribute greatly to Harry's domestic happiness. One of her peculiarities consisted in not retiring to rest at the usual hour. She would frequently employ half the night in examing the wardrobe of the family, to see that nothing was missing, and that everything was in its proper place. The following is told as a proof of her oddities. One morning, about three o'clock, having been unsuccessful in a search, she awoke Mr. Erskine from a sound sleep by putting to him this important interrogatory, "Harry, lovie, where is your white waistcoat?"

